# Duanzi as Networked Practice: How Online Satire Shapes Psychological Well-Being, Social Support, and Issue Knowledge for Chinese with Different Social Capital during COVID-19 Outbreaks

**DOI:** 10.3390/ijerph18189783

**Published:** 2021-09-17

**Authors:** Ji Pan, Gang (Kevin) Han, Ran Wei

**Affiliations:** 1 Center for Information and Communication Studies, Journalism School, Fudan University, Shanghai 200433, China; panji@fudan.edu.cn; 2 Greenlee School of Journalism and Communication, Iowa State University, Ames, IA 50011, USA; 3 School of Journalism and Communication, Chinese University of Hongkong, Hong Kong; ranwei@cuhk.edu.hk

**Keywords:** COVID-19, duanzi, social capital, health communication

## Abstract

Practices oriented to digital technologies are being invented to change how people cope with crises. This study examines how Chinese netizens’ networked practices (e.g., liking, sharing, or commenting) with COVID-19 related duanzi (short online satires) influenced their psychological well-being, external social support, and issue knowledge during the pandemic. The role of social capital in moderating these relations is explored. Findings from the survey demonstrate that the act of “liking” a COVID-19 duanzi on WeChat has become a routine practice for Chinese netizens to kill time during the quarantine. However, the more bonding social capital one already had, the less they depended on duanzi “liking” to kill their boredom. Those less supported outside the family household, or less knowledgeable about the virus were also more likely to share a COVID-19 duanzi. Bonding social capital promotes one’s well-being, therefore, the positive psychological effect of duanzi sharing or commenting grows more pronounced for netizens with more bonding social capital. Bridging social capital brought external social support. Netizens with more bridging social capital obtained more external support and more COVID-19 knowledge from duanzi sharing. The theoretical and practical implications are elaborated in the conclusions.

## 1. Introduction

Coronavirus disease 2019 (COVID-19) appeared in Wuhan in December 2019 and spread rapidly afterward. The COVID-19 outbreak stands out as China’s first national health crisis in the mobile social media era [1]. Chinese authorities took public health measures including intensive surveillance, epidemiological investigation, or the full lockdown of cities to prevent the infectious disease. The lengthy lockdown following the successive outbreaks forced an unprecedentedly large number of Chinese to stay home with their Internet and digital devices. During the lockdown, public spaces such as restaurants, museums, parks, libraries, and schools in major Chinese cities were closed. The local government deployed technologies like networked surveillance cameras and health codes to compel or persuade people to stay at home. At the height of the pandemic, social contact outside one’s core family household was minimized. Most Chinese had to cope with the anxiety from potential exposure to a largely unknown virus in a prolonged period of social isolation. Social isolation affects how individuals cope with stress from the pandemic, which has changed their lives drastically (e.g., [2,3,4]). Scholars have warned that one’s response to and recovery from the pandemic can be hampered by deficiencies or disruptions in social capital, or resources from social networks [5,6], brought about by mandatory physical distancing [7].

However, the wide penetration of social media, WeChat (In 2011, Tencent released the mobile messaging application Weixin (“micro-message”) or WeChat. It was rapidly augmented with social networking functions such as the “friend circle” content feed, and the possibility of following the “public platform” official accounts run by brands, organizations, or news outlets. Other added functions included WeChat Pay, enabling users to perform mobile payments, City Services, a system for public and private public services, and WeChat Search, a proprietary in-app search engine), in particular, into the everyday life of Chinese households has profoundly changed what people can do and how they may feel when home-bound throughout the pandemic. WeChat and its likes have radically changed how different people cope with crises. In the 2003 SARS epidemic, information was still mainly offered on notice boards in residential compounds [8]. In contrast, WeChat has now become the most popular information sharing platform and the most accessible means of social engagement outside one’s family household during COVID-19 outbreaks. In fact, social media use in China skyrocketed during the COVID-19 outbreaks—millions relied on WeChat for COVID-19 information, external social support, or some fun during the lockdown [9]. Characterized by a massive usage scale, indispensability in daily life, and a plethora of nested public services [10], WeChat’s technological features enable individual users to engage in a variety of cultural and social practices without leaving their family household.

Among the various practices supported by WeChat, the creation, reproduction, and dissemination of short online satires, in textual or multimedia form, constitutes a staple of Chinese cyber culture [11]. It is especially so when “duanzi”, or snappy jokes with a biting sense of humor and light-heartedness [12], flooded WeChat as a prominent form of grass-root expression. Sharing duanzi has become a wildly popular social activity since the late 1990s when mobile phones and the Internet started to boom in China [11]. The results of an online survey People’s Tribute Survey Center conducted in 2010 with 8762 netizens and a written questionnaire of 1048 respondents concluded that “Everybody in China does duanzi”. The popularity of duanzi lingers to this day. The wildfire circulation of duanzi has become a distinctive phenomenon in China’s cyberspace throughout the COVID-19 pandemic as netizens read, spread, or created short satirical messages to express themselves. For example, home-bound Chinese netizens created, shared, and commented on COVID-19 duanzi to joke about the effectiveness of Shuanghuanglian (a traditional Chinese medicine allegedly effective for COVID-19); ridicule the endlessness of the lockdown; or mock the wearing of facemasks in public space (see Table 1 for a categorization of duanzi). Many routine practices including commenting, sharing, or “liking” a duanzi have emerged from the circulation of duanzi messages, activating the emotional, informative, and social resources inherent in one’s existing social networks [13]. Contingent on one’s technological, social and cultural conditions, media practices related to duanzi hiked during COVID-19, while most offline means for the same purposes evaporated overnight [14].

Nevertheless, most extant literature have used discourse or textual analysis to examine duanzi’s substantive content from the perspective of political resistance [15,16]. Very few have explored duanzi as a set of networked practices, conceptualized as routinized media-related acts supported by shared understanding, rules, emotion, ends, projects, and beliefs [17,18]. Through duanzi’s online circulation, this set of related practices may generate multiple social networks, whose significance goes beyond political resistance [11]. Even fewer have investigated how bonding and bridging social capital moderates the relations between duanzi practices and one’s subjective knowledge, acquisition of external social support, or psychological well-being when all of the above were scarce throughout a global pandemic.

In response, this study draws on health communication and social capital literature to explore how an individual netizen’s media practices with COVID-19 related “duanzi” on WeChat (e.g., liking, sharing or commenting) relate to their psychological state, acquisition of external social support, and subjective knowledge about the virus. Duanzi is conceptualized as a series of routinized social media practices with satirical humorous contents when alternative modes of socialization withered in the pandemic. Furthermore, we illustrate the moderating role of bonding and bridging social capital on the relations between duanzi practice and its psychological, knowledge, and social outcomes. We add to the literature by demonstrating that duanzi practices support critical boredom-killing rituals for netizens wanting bonding social capital or afford netizens to signal for the lack of knowledge or external social support during China’s COVID-19 pandemic—bridging (bonding) social capital mainly boosts the social (psychological) impact of these practices.

## 2. Literature Review

### 2.1. Duanzi as Networked Practices

Duanzi is a unique form of digital media that has gained immense popularity in China’s cyberspace. Developed from China’s traditional art form of xiangsheng (or crosstalk), duanzi refers to jokes characterized by lightness and often times a biting sense of humor. Spreading like wildfire in cyberspace, duanzi has now become a key means for Chinese netizens to express personal views on public affairs; to entertain each other; or to show off their personal wisdom [19]. Despite tightened control over cyberspace [20], the de-centralized nature of digital networks and Chinese President Xi Jinping’s emphasis on “mass line through the Internet” (网络群众路线 in Chinese) have made it possible for netizens to participate in the production and dissemination of duanzi [21,22,23,24].

Scholars have examined the significance of duanzi contents [15,16]. They distinguished between hyperbole, jocularity, and understatement as sub-categories of online irony [25]. Some found parodies, jokes, slippery jingles (顺口溜 or Shunkouliu), verse, songs, flash videos, and spoof (恶搞 or E’gao) to be major forms of satire in Chinese cyberspace [12,16,19,26]. In particular, Nordin and Richaud [27] analyzed the satirical power of “Cao Ni Ma” (草泥马; Grass Mud Horse) as a code widely deployed by Chinese netizens to subvert official discourses. Tai [28] examined the playful protests around the icon of a “Hexie” (河蟹 or river crab as a code for censorship). Yang and Jiang [11] distinguished between red, gray, and yellow duanzi (referring respectively to jokes about mainstream ideology, social political criticism, or about sex), all serving as forms of networked practice in China. Nevertheless, reducing duanzi to content may ignore its potential effect on netizens in dynamic networks of digital communication, or its social implications in a network society [29].

Others have taken a more practical approach and treated duanzi as a series of networked socio-cultural practices [11,30]. The practical approach dates back to earlier studies on the unintended health effect of television. Rather than focusing on televised contents, scholars found that it was the snacking and sitting during television consumption that affected a viewer’s health (e.g., [31,32]). Likewise, Yang and Jiang [11] maintained that the online communication of satire builds social networks via an individual’s online cultural expression such as duanzi, sentence-making, or multi-media remixing practices. Without entirely excluding the substantive contents of communication from analysis, scholars maintained that duanzi as a form of mediated communication is ready-to-spread on digital networks. In its diffusion process, a popular duanzi may bring forth a set of real-time collaborative (re)production, circulation, or networked meaning, re-assembling practices by connected netizens on social media [26].

By deduction, the media practices that emerge from duanzi’s online circulation among Chinese netizens may not only support novel modes of self-expression within one’s online social networks. Furthermore, duanzi practices can otherwise relate physically distanced individuals via the communication of humorous and light-hearted contents at a time of heightened uncertainty and anxiety. Thanks to the online dissemination of duanzi during the COVID-19 outbreaks, connected netizens may communicate about public issues related to the disease in a more light-hearted, non-hierarchical, and networked fashion. These duanzi-based communication practices carry social, informative, and psychological consequences. Accordingly, this study conceptualizes duanzi as distinct forms of networked practice that are related to satirical jokes about COVID-19. Drawing on the work of Yang and Jiang [11], we operationalized duanzi practice as an individual’s routinized acts of liking, sharing, or commenting on COVID-19 related duanzi messages on WeChat during the pandemic outbreaks.

### 2.2. Social Connection Benefits of Duanzi Practices

Media practice carries social connection benefits. In the 1970s, Wurtzel and Turner [33] found that telephoning practices offered callers ready social connectedness by supporting immediate interpersonal interactions. Routinized telephoning builds one’s “psychological neighborhood” as a socially supportive and information-sharing community. On the Internet, the diversity of media practice that may boost one’s social connectedness has increased rapidly. The convenience, connectivity, and social cues of digital network support a range of synchronous or asynchronous interactions with different ties—from families to communities of interest or of practice [34]. As social media enable individuals to manage relationships with different social connections and lower the barriers for exchange of social support, researchers have argued that social media help individuals tap into latent social capital resources in their network [35,36]. 

In particular, a functional approach—exploring specific types of media practice rather than general media use—helps explicate the impact of digital technologies on an individual’s perception of social connectedness. Internet scholars have stated that like other religious or social rituals, participation in the active creation and communication of online content serves solidarity functions [37]. Specifically, the act of participating in massive multi-player online (MMO) games offers the experience of relatedness, motivating individuals to play again. Kellie [38] explained that playing MMO or AR online games creates a virtual “third place”, where players may produce their own contents, comment on, or converse with a much wider variety of contacts. Likewise, the acts to create and/or publish updates or comments on social media expands a netizen’s access to a broader range of social ties and enable second-degree social connections that are important for the generation of social support from outside one’s family household [36]. Posting comments on a friend’s social media content enables users to interact with the poster’s extant social network as opposed to their own, which potentially facilitates broader social connections outside one’s existing strong-tie network [35,36].

Furthermore, the act of sharing one’s personal opinion, individual activities, or creative works with a growing number of social media “friends” may not only expand but also strengthen one’s personal social connections. For instance, the netizens’ act of sharing their personal requests on Facebook has been found to create more bridging social capital, leading them to see Facebook as a better source of networked communication than if they do not do so [35,36]. Related, Steinmuller [39] stated that the frequent sharing of light-hearted satires such as duanzi between netizens in cyberspace gives rise to multiple “communities of complicity”. Comparable to telephoning, the dynamic network of duanzi sharing could bring forth a broader “psychological neighborhood” that affords a sense of social connectedness and social support therein [33].

Additionally, the practice of sending a “like” kudo to friends on social media, which indicates a small and rather low-cost signal of attention to one another, also supports relationship maintenance [35]. The “like” function of most social media platforms has been a key feature to support relationship maintenance. Scholars have posited that social media functions such as News Feed or “like” buttons facilitate light-weighted netizen interactions that may support social capital processes with little effort. The ease and low cost of exchanging “likes” on social media platforms suggest that this practice tends to occur quite frequently in both weak-tie and strong-tie networks. For instance, norms of reciprocity in the exchange of “likes” among connected netizens suggest that the circulation of “like” as a token of mutual recognition and attention may evolve into a social game, which keeps or strengthens social connections between a wide range of online friends [36].

How does a netizen’s routine practice to share, “like”, or comment on a duanzi message promote their perception of broader social connectedness and social support during the pandemic? For this inquiry, the distinctive features of COVID-19-related duanzi, especially its light-hearted humor and ready-to-spread form, add to the social impact of its online circulation during the COVID-19 outbreaks. It is especially so when anxiety and uncertainty about the virus both rose high, while offline social interactions with existing bonding or bridging ties outside one’s family household vanished abruptly. By deduction, an individual netizen’s practice to share, “like”, or comment on a viral COVID-19 duanzi may promote one’s sense of “being in a community” and activate one’s social networks established prior to the pandemic [11]. In particular, the acts to “like”, share, or comment on an online duanzi may serve to produce a communal laughter; affirm the value of interactants; show attention and care for (potential) victims; distribute COVID-19 related knowledge; or even show off an individual’s linguistic or literary talents while other modes of social interactions disappear overnight. Therefore, we hypothesize:

**Hypothesis** **H1.** *The acts of sharing, liking, or commenting on a duanzi are positively related to a respondent’s perceived (a) social connectedness and (b) social support from outside their households.*

### 2.3. Positive Psychological Outcomes of Duanzi Practices

How people act toward media brings psychological outcomes. In the 1970s, scholars found that the routine practice of telephoning helped reduce one’s sense of boredom, isolation, and anxiety—regardless of the content of phone conversations [40]. Likewise, people turned to radio listening or television viewing in response to daily hassles and stresses [41,42], though they did not always select mood-enhancing contents [43]. The act of media consumption itself suffices to generate remarkable benefits for well-being by producing positive effects, relaxation, or stress reduction [44,45,46]. Scholars have explained that netizens’ Internet use promoted health and well-being by showcasing their autonomy to connected acquaintances [47,48]; by enhancing the retention of health-related knowledge [49]; or sometimes by gratifying their pressing needs for aids or for tension-release [50].

What are the positive psychological outcomes of COVID-19 duanzi practice? For duanzi practices through the COVID-19 outbreaks, when netizens share or write about their personal experience, strong emotion or careful thoughts about this rather stressful health crisis within or around a duanzi, the stressed tend to re-appraise the situation, leading to remarkable psychological or knowledge improvements [51,52]. A cognitive, emotional and a behavioral explanation have been offered. Some posited that the acts of producing, consuming, or sharing humorous content such as duanzi messages online may equip an individual with a more optimistic cognitive framework, leading one to assess an otherwise negative situation in a somewhat more positive light [53]. Alternatively, the processes of sharing, liking, or publishing personal comments on a duanzi help lower one’s emotional arousal associated with stressful events, translating into a less negative psychological experience of the COVID-19 outbreak as stressors [54]. Specifically, health communication scholarship established that reading or writing about humorous content (like duanzi) may reduce one’s sense of shame in otherwise embarrassing health situations, making the experience feel more positive [55,56]. In such scenarios, difficult health behaviors (e.g., testicle checks or checks involving nudity) are more likely to happen, often resulting in increased subjective or objective knowledge about a condition.

Accordingly, the everyday media practice to share, like, or comment on humorous duanzi messages during the COVID-19 outbreaks can support home-bound netizens to communicate about the pandemic in a creative and playful tactic [57]. Based on the literature, such tactics may lead to a more positive state-of-mind, less boredom, and more subjective knowledge about the virus. Hence, we hypothesize:

**Hypothesis** **H2.** *The acts of sharing, liking, and commenting on a duanzi are positively related to a neitzen’s (a) psychological well-being, (b) reduced boredom, and (c) COVID-19 related knowledge.*

### 2.4. Bonding Social Capital as a Moderator

How does bonding social capital moderate the impact of duanzi practice? Since Durkheim’s [58] seminal writings on suicide, social capital and its correlates have been repeatedly linked to health outcomes [59,60]. Social capital comprises resources in social networks to be accessed or mobilized in purposive actions [5,6]. It predicts psychological state [61,62]; participation in physical activities [63]; or access to health services [64]. Online social capital generates instrumental (e.g., health knowledge, tangible support) and expressive returns for health (e.g., life satisfaction, well-being) [65,66]. The significance of social capital grows in COVID-19 outbreaks as people in prolonged social isolation demand more solace and certainty [7].

Bonding social capital mainly resides in intimate inward-looking strong ties in smaller, exclusive circles of family or close friends. Empirical inquiries suggest that a wide range of social media practices that boost one’s self-image in strong-tie networks can mobilize or generate bonding social capital [36,67]. For support, disclosure of one’s self-image in cyberspace facilitates both online and offline close relationships [35,68,69,70], resulting in a boost of bonding social capital. Likewise, Burke [71] found that compared with the passive consumption of others’ information in cyberspace, the act of participating in online content production has a stronger association with bonding social capital. By deduction, media practices to present a positive self-image in online networks by participating in the production of various networked contents may promote one’s bonding social capital.

For this study, the routine media practice to “like” or to comment on a published duanzi enables netizens to boost their bonding social capital during COVID-19 outbreaks. In particular, publicly commenting on a humorous and satirical message in cyberspace may promote one’s perception of self-image. As netizens publish rounds of detailed personal comments to a COVID-19 related duanzi, they could be expressing their personal judgment about the pandemic; showing off their sense of humor; or sometimes demonstrating their superior intellectual and literary creativity in multiple networks of connected acquaintances. Moreover, an individual’s act to exchange “likes” about duanzi makes a shallow but active and positive sign of mutual recognition in a context when social recognition from outside one’s family household became rare. In effect, both receiving or sending a “like” token to each other’s’ duanzi can improve one’s perception of self-image or one’s image presented to others in online networks. Furthermore, unlike the authoring of a viral duanzi, the acts of clicking a “like” button or commenting on a published duanzi are more likely to be noted and recognized by contacts in one’s strong-tie network than by broader acquaintances. By doing so throughout the pandemic, netizens can make themselves appear more attractive, talented, or knowledgeable to their online or offline close relationships, motivating others to form and maintain strong bonding social ties with them [68].

Once mobilized by duanzi practices (such as liking or commenting in our context), bonding ties can often “ensure chicken soup when you are sick” [72]. In other words, bonding social capital offers people in need or in stress both psychological and social benefits. Socially, extant scholarship shows that bonding social capital enhances one’s feeling of being sufficiently supported by intimate others; brings physical succor or other larger benefits such as the willingness of bonding ties to loan a substantial sum of money to those in need. Psychologically, scholars claim that more bonding social capital may help reinforce one’s self-esteem, reduce one’s feeling of loneliness and boredom [73], promote psychological adjustment to stress, or strengthen one’s sense of personal control in the form of higher self-efficacy [74]. Regardless of intent, the act of engaging in affectionate interactions within bonding-tie networks suffices to exert a tangible effect on the alleviation of stress symptoms following exposure to an acute stressor [75]. According to Erickson [73], “People are healthier and happier when they have intimates, who do care about and for them” (p. 26).

In accordance, bonding social capital lies between a netizen’s routine practice of “liking” or commenting on an online duanzi and its positive social or psychological outcomes. It is especially so during the COVID-19 outbreaks. At the height of the COVID-19 outbreaks, most people were prohibited from building new offline social connections, the health consequences of stress, monotony, and social isolation were aggravated, and resources in various strong-tie networks become even more important for coping [11]. Therefore, we hypothesize:

**Hypothesis** **H3.** *Bonding social capital moderates the relationship between acts of liking and commenting on a duanzi and a respondent’s (a) psychological well-being, (b) social connectedness, and (c) reduced boredom.*

### 2.5. Bridging Social Capital as Moderator

What is the role of bridging social capital as a moderator? Bridging social capital includes resources from one’s weak ties, who share no intense emotional attachment, but know each other and will share information, co-operate, or assist one another if needs be [72]. For netizens who seek community experience, the creation and maintenance of larger diffuse weak-tie networks have become cheaper and easier thanks to social media [76]. Ellison et al. [35] speculated that by supporting a wider range of networking activities, social media encourage netizens to activate latent social ties in their networks, transforming these into weak ties associated with bridging social capital gains.

Among the many emerging social media practices, the act to pass along interesting content among weak-tie contacts often forges a communal experience that promotes bridging social capital. Empirical studies have found that social media activities such as photo sharing, file-sharing in group chatting, or even social searching encouraged netizens to reach out to distant acquaintances who may share common interests [77,78]. When most in-person contact with a broader range of distant acquaintances was banned during the COVID-19 outbreaks, netizens may have depended even more on social media sharing rituals to overcome the limits of space and time and to re-gain a sense of “being in community”. Sharing behaviors particularly promote bridging social capital, as scholars have found that passing along fun and humorous content in cyberspace is more suited to forming shallower social relationships than strong ties [79]. Therefore, sharing a COVID-19 related duanzi among one’s acquaintances during the epidemic may constitute a major ritual for home-bound netizens to create and maintain diffuse networks of bridging social capital [34,78,80].

In effect, bridging ties constitute a major source of informational and other social support benefits, especially during crises like the epidemic [73]. Erickson [73] claimed that people tended to “do much better” and acquire more health-related information and knowledge if they were connected to larger and more diverse social networks outside their close circles (p. 25). To explain, bridging social capital supports easier access to health-related knowledge from distant connections and/or from a much larger diversity of viewpoints [81]. Moreover, people in extensive weak-tie networks often perceive less risk in disclosing useful information to one another, offer more objective feedback in communication, and require less role obligation to reciprocate information or assistance [82]. For instance, the progress of science itself has come via the crossing of larger networks of dispersed groups and individuals sharing information and methods with one another [83]. In addition, bridging social capital can bring social support for various purposes from the right contacts outside one’s close circle. Scholars have found that people with more bridging social capital usually have better chances of changing jobs or finding new positionss in a highly competitive marketplace [84,85]. The knowledge and social support benefits of online bridging social capital become critical in a major public health crisis like COVID-19. Rains and Keating [86] found that the health bloggers who received the least support from close friends and family members benefited the most from social supports from weak-tie stranger blog readers. Likewise, a study of tsunami-hit Indian communities proved that bridging social capital played a key role in reducing resource disparities between communities, and to enhance the sharing of key information/knowledge throughout the emergency [87].

Due to massive social isolation and physical distancing during the COVID-19 outbreaks, strong ties outside one’s family household may not be able to provide sufficient social or emotional support as they normally do. For the same reason, diverse sources of COVID-19 related information were in high demand. Against the backdrop, we hypothesize:

**Hypothesis** **H4.** *Bridging social capital moderates the relation between duanzi sharing practices and a respondent’s (a) COVID-19 knowledge, and (b) social support from outside one’s household.*

## 3. Method

### 3.1. Data and Sampling

An online survey was conducted in March 2020 in Shanghai, China’s largest city of 24 million. We chose to collect data from Shanghai for several reasons. First, the Internet penetration rate (74.1%) in Shanghai is among the highest in China [88]. Second, Shanghai was close to the epicenter of the COVID-19 outbreaks, but far enough for uncertainty to rise, for the public to brace themselves, and for duanzi messages to spread while citizens stayed home.

Respondents were selected via a quota sampling procedure. Wenjuanwang (www.wenjuan.com, 问卷网 accessed on 26 July 2021), a reputed local online survey research company in Shanghai, was hired to execute the survey. Wenjuanwang owns proprietary panels by which a company obtains participants, reaching them on devices including desktops, laptops, smart phones, and tablets. For quota sampling, the survey company was instructed to randomly draw from their proprietary panel 1100 potential respondents, whose makeup represented the demographics of permanent residents with Internet access in Shanghai. With the Institutional Review Board’s approval, data collection was carried out from March 12 to 20, 2020. After deleting incomplete or invalid questionnaires, 1000 responses were received. The completion rate reached 90.9%.

Among all respondents, 52.4% were male, 47.6% female. In terms of age, 33.8% were 24 years old or younger, 46.1% 25–40, and 20.1% 41 and older. For education level, 20.1% of respondents finished middle school or lower, 32.7% went to a high school, technical secondary school, or vocational school, and 47.2% attended college, university, or received a higher degree. Their monthly income were categorized as 5000 RMB or below (37.4%), 5000–10,000 (33.2%), 10–20 thousand (17.6%), and 20,000 or above (11.8%), respectively. This sample profile matches the parameters of the Internet user population in Shanghai.

Before filling out the questionnaire, respondents were instructed to read through a list of examples for different types of COVID-19 related duanzi that were most popular in cyberspace during the pandemic. This measure and a question following these examples confirmed that all respondents had read some COVID-19 related duanzi during the epidemic outbreaks and they could recognize one when they stayed at home during the quarantine.

### 3.2. Measures

Duanzi practice was measured with a set of 5-point Likert scale items. Respondents were asked whether they agreed (1 = strongly disagree, 5 = strongly agree) with the statements concerning three aspects: “like” (two items: “I usually give ‘likes’ to; or save, the COVID-19 duanzi messages that I read, watched or viewed”, *alpha* = 0.67, M = 2.88, SD = 0.92); “sharing” (three items: “I usually forward the COVID-19 dunazi messages that I read, watched or viewed to friends, chat group, or to WeChat Moments”, *alpha* = 0.80, M = 2.77, SD = 0.96), and “commenting” (two items: “I usually comment on the COVID-19 dunazi messages that I read, watched or viewed and share with my friends, or to chat groups”, α = 0.80, M = 2.50, SD = 1.04).

Bridging social capital was gauged with five 5-point Likert-scale items validated by Gil de Zúñiga et al. [89], Skoric et al., [90] and Williams, [91], asking respondents whether they agreed (1 = strongly disagree, and 5 = strongly agree) with the following statements: “Interacting with people online makes me feel connected to the bigger picture; interacting with people online makes me interested in what people unlike me are thinking; I am willing to spend time to support general online community activities; interacting with people online makes me want to try new things; and interacting with people online makes me feel like part of a larger community” (α = 0.75; M = 3.70, SD = 0.65).

Bonding social capital was assessed with six 5-point Likert-scale items adapted from Gil de Zúñiga et al. [89], Skoric et al., [90] and Williams, [91]. Respondents were asked whether they agreed with the following statements (1 = strongly disagree, and 5 = strongly agree): “There is someone online I can turn to for advice about making very important decisions; the people I interact with online would share their last dollar with me; the people I interact with online would put their reputation on the line for me; I do not know people online well enough to get them to do anything important; the people I interact with online would help me fight an injustice; and, there are several people online I trust to help solve my problems” (α = 0.85; M = 3.16, SD = 0.82).

Psychological well-being was gauged with a 5-point Likert scale. Respondents were asked whether they agreed (1 = strongly disagree, and 5 = strongly agree) with the following sixteen statements, partially adapted from Ryff et al. [92], about feelings or emotions related to COVID-19: “I feel happy; I’m pleased with myself; I feel energized; I feel calm; I feel secure; I feel content; I can make decisions with ease; I’m sedate; I’m confident; I’m not upset; I’m competent; I believe I can overcome difficulties; I don’t worry too much about things that aren’t necessary; I am not disturbed by unimportant things; I let go those disappointing things; I’m not nervous or upset when thinking about recent concerns” (α = 88; M = 3.47, SD = 0.59).

For social connectedness, respondents were asked how often they felt the following thirteen psychological states, partially adapted from the UCLA Loneliness Scale (1 = not at all, and 5 = always): “I am not ‘in tune’ with the people around me; I lack companionship; there is no one I can turn to; I feel alone; I don’t feel part of a group of friends; I feel that I am no longer close to anyone; my interests and ideas are not shared by those around me; I feel left out; my relationships with others are not meaningful; no one really knows me well; there are no people who really understand me; I feel shy; people are around me but not with me” (α = 92; M = 3.65, SD = 0.80).

For reduced boredom, respondents indicated their agreement on a 5-point Likert scale (1 = strongly disagree, 5 = strongly agree) with seven statements: “Time was passing by slower than usual; I was stuck in a situation that I felt was irrelevant; Everything seemed repetitive and routine to me; I felt bored; I seemed to be forced to do things that have no value to me; I wished I were doing some-thing more exciting”. Items were adapted from Fahlman et al. [93] and reverse coded for further analyses (α = 0.77; M = 3.11, SD = 0.80).

COVID-19-related knowledge was measured by four 5-point Likert scale statements concerning one’s subjective knowledge about the virus including: (1) I know pretty much about the coronavirus; (2) Among my circle of friends, I am one of the “experts” on the coronavirus; (3) Compared to most other people, I know less about the coronavirus; and (4) I feel very knowledgeable about the coronavirus. The statements were adapted from Goldsmith [94] and Flynn and Goldsmith [95]. We reverse coded the third item in analysis. A composite index was constructed by averaging the combined item, with higher scores indicating more knowledge (α = 0.84; M = 2.94, SD = 0.76).

Social support from outside the family household was assessed with 5-point Likert scales adapted from Zimet, Dahlem, Zimet, and Farley (1988). Respondents indicated their agreement (1 = strongly disagree; 5 = strongly agree) on four statements such as “My friends really try to help me; I can count on my friends when things go wrong; I have friends with whom I can share my joys and sorrows; I can talk about my problems with my friends” (α = 0.68; M = 3.73, SD = 0.64).

Demographics including age and gender were analyzed as control variables (1 = male, 0 = female), education (1 = least educated, 5 = most educated; M = 3.5, SD = 1.18), and household income (1 = least income, 5 = most income; M = 3.69, SD = 1.04).

## 4. Findings

### 4.1. Data Analysis

A series of linear regression analyses with PROCESS module in SPSS were performed to test the set of hypotheses. First, data analysis assessed the correlations between online duanzi practices (e.g., liking, sharing, and commenting) and positive psychological outcomes, external social support, and subjective issue knowledge.

Afterward, the interaction items in regression analyses were scrutinized to test the hypotheses about the moderating effect of bonding and bridging social capital on the relations between duanzi practices and the dependent variables.

### 4.2. Direct Effect of Duanzi Practices

H1 was not supported. The findings in Table 2 and Table 3 show that none of the duanzi liking, duanzi sharing, or duanzi commenting practices was significantly related to the netizen’s perception of being socially connected during the pandemic (H1a). Most duanzi practices do not seem to add to a netizen’s sense of being connected during the quarantine. Contrary to H1b, the act to share a duanzi among WeChat friends is even negatively related to the amount of social support one felt from outside one’s family household (β = −0.28, *p* < 0.05). Overall, the social connection benefits of duanzi practices during the COVID-19 outbreaks were not significant.

Neither H2a nor H2c were supported, but H2b was partially supported. In particular, the findings in Table 3 demonstrate that none of the acts of sharing, liking, or commenting on a duanzi message was positively related to a neitzen’s psychological well-being. However, the practice to “like” a duanzi message published by a social media contact was positively related to boredom reduction (β = 0.29, *p* < 0.01). In other words, though various practices related to duanzi were not overall significantly related to one’s psychological well-being, the routine act of “liking” a duanzi did help kill some boredom for home-bound netizens during the pandemic. Sending “likes” to a duanzi posted online proved to be an obviously effective means to seek fun and to feel less bored during the lengthy quarantine. Additionally, analyses showed that the acts of sharing (β = −0.31, *p* < 0.05) and that of commenting on a COVID-19 duanzi message (β = −0.40, *p* < 0.01) were negatively related to the subject knowledge that a netizen felt that they already had about the pandemic.

Finally, bonding social capital proved to be a consistent and potent predictor of psychological well-being during the pandemic (β = 0.14, *p* < 0.05), though its contribution to social connectedness and boredom reduction was negligible. In comparison, bridging social capital brings more social support from outside one’s family household (β = 0.30, *p* < 0.01), but exerts little impact on COVID-19 related knowledge.

For the moderating effect of bridging and bonding social capital, the interaction items in linear regression models were examined.

For H3, findings showed that bonding social capital moderated the relations between the act of liking a duanzi and boredom reduction (β = 0.07, *p* < 0.05). In particular, the more bonding social capital one already has, the less the act of “liking” a duanzi may contribute to one’s boredom reduction. Bonding social capital also moderates psychological well-being’s correlation with the acts of sharing a duanzi (β = 0.04, *p* < 0.05), or commenting on one (β = 0.05, *p* < 0.05). The positive psychological effect of sharing or commenting on a duanzi grows more pronounced for those blessed with more bonding social capital. Nevertheless, the impact of the interaction items on social connectedness was insignificant. In general, the effect of bonding social capital was most obvious on psychological well-being when people were forced to stay home for the pandemic.

For H4, findings demonstrated that bridging social capital moderates the relations that duanzi sharing has with one’s external social support (β = 0.09, *p* < 0.01) and with COVID-19 related issue knowledge (β = 0.12, *p* < 0.001). Besides, bridging social capital also moderated the relation between duanzi commenting and one’s COVID-19 knowledge (β = 0.14, *p* < 0.0001). Netizens with relatively more bridging social capital tended to obtain more external social capital as they shared a duanzi message during the COVID-19 outbreaks. In addition, the benefit in subjective COVID-19 knowledge that one may obtain from the acts of duanzi commenting or sharing were more substantial for netizens who had more bridging social capital.

## 5. Conclusions

To conclude, this study found that the act of “liking” a COVID-19 duanzi on WeChat has become a routine practice for home-bound Chinese netizens to relieve their boredom during the lockdown. The more bonding social capital one already had, the less they depended on the game of duanzi “liking” for boredom reduction. None of the duanzi sharing, liking, or commenting practices added directly to one’s sense of social connectedness or psychological well-being. In contrast, those less supported outside the family household or less knowledgeable about the virus were more likely to share a COVID-19 duanzi with others. Bonding social capital promotes one’s well-being: the positive psychological effect of duanzi sharing or commenting grew more pronounced for people with more bonding social capital. Meanwhile, netizens with more bridging social capital obtained more external support and more COVID-19 knowledge from regular duanzi sharing. Knowledge gains from duanzi sharing or commenting practices grew higher for those with more bridging social capital.

The consistent effect of duanzi “liking” suggests that exchanging shallow tokens of approval about a light-hearted duanzi was a critical ritual for Chinese netizens to kill time and have some fun during the pandemic. Extant scholarship suggests that the consumption of entertaining or funny content may lower emotional stress and relieve the sense of loneliness brought up by a lockdown, leading to a more positive psychological experience [54]. We added a dimension by showing that apart from the consumption of humorous contents, the recurrent ritualistic practice of proclaiming one’s symbolic “liking” for a duanzi published by an existing WeChat contact may become a convenient mode of social interaction that helped resist the monotony of quarantined life. To explain, during the COVID-19 outbreaks when anxiety and social isolation were at their highest, exchanging kudos for humorous content may not make people feel more connected or psychologically better-off. Vis-a-vis the unprecedented global crisis, especially the grave uncertainty and suffocating social isolation, rather shallow forms of duanzi-related social media practices may not suffice in affording a strong sense of community. Their stress-relieving effect is limited against the overwhelming crisis. Nevertheless, “liking” each other’s duanzi could still become a social game in itself—Chinese netizens may follow or even actively compete with one another on WeChat to give “likes” as a gesture of mutual recognition or to receive “likes” as an indicator of their popularity. The act of regularly receiving or sending symbolic approval among contacts who could not meet face-to-face significantly relieved the boredom of the lengthy quarantine. “Liking” a COVID-19 duanzi has become a ritual as well as a competitive social game. Furthermore, the moderation effect of bonding social capital demonstrates that netizens with less bonding social capital may kill more boredom by “liking” an online duanzi. In other words, “liking” each other’s duanzi on social media makes a much more effective means of boredom reduction, especially for those surrounded by less bonding ties during the pandemic. Viewed together, the media ritual of duanzi “liking” might offer a novel way for Chinese netizens to compensate for the lack of bonding social capital as a critical but ready source of fun and joy throughout the lockdown.

The finding that netizens less supported outside their family household or feeling less knowledgeable about the virus were more likely to share a duanzi seemingly contradicts the hypotheses. Contextualized in the COVID-19 pandemic, however, the finding carries multiple implications. First, it suggests that the act of sharing a duanzi may not add directly to one’s subjective knowledge or to the amount of social support one obtained from outside the family household. Communication about duanzi brings more fun than substantive knowledge or external social support. By implication, when satires were circulated in crises rife with uncertainty and anxiety, the impact of its digital form and the ritual of its dissemination might far outweigh the effect of its substantive contents on knowledge acquisition. In contrast, the findings suggest that when rumors about the pandemic were rampant earlier in its outbreak, netizens less knowledgeable about the virus were also less prepared to discern or acquire hardcore scientific knowledge about the disease. However, caught in the crisis, they needed to somehow “talk about” the pandemic as all others were apparently doing so without revealing their lack of knowledge. Therefore, they were more inclined to spread or talk about COVID-19 duanzi in a light-hearted way than their more knowledgeable peers. Furthermore, the act of sharing a duanzi may also be interpreted as an outcry from less resourceful netizens to seek COVID-19 information or social support from a broader network, both of which were scarce during the lockdown. When offline social interactions with weak-tie contacts were minimized, passing along a funny duanzi may become a harmless and socially appropriate means to activate or reach out to bridging ties in a relatively loose online network. In fact, the finding that bridging social capital moderates the relation of duanzi sharing with social support and with subjective issue knowledge lends further confidence to the “activating” effect of duanzi sharing. Detailed examination of findings supports that bridging social capital not only exerts a positive direct effect on external social support, but also reverses the negative correlation that duanzi sharing originally has with external social support or with COVID-19 knowledge. Viewed together, the findings imply that during the pandemic, the act of duanzi sharing or duanzi commenting can have distinct meanings for netizens with or without sufficient bridging social capital. For those with plenty of bridging social capital, passing along a humorous duanzi during the lockdown can activate and convert existing social capital into more COVID-19 knowledge or substantive social support from broader weak-tie networks. For those with less bridging social capital, the same duanzi sharing practice mainly served as compensation for the temporary lack of socialization or as a signal for the lack of information about the raging pandemic.

Finally, the impact of bonding social capital on psychological well-being supports its benefit for stress coping. Having many caring intimates around does matter for health and happiness throughout the pandemic [73]. However, our findings add to the literature by demonstrating that bonding social capital has no direct effect on boredom reduction or on one’s social connectedness. To explain, people who were home-quarantined during the COVID-19 outbreak were forced to stay with one’s core family household for an unprecedentedly extended period of time. Aggravated by massive social isolation and distancing from the outside world, information redundancy and monotony may soar over time within the family household. Instead of bringing fun or strengthening one’s felt social connectedness, the constant and exclusive presence of a smaller circle of strong-tie contacts day in and day out may even remind people of the abrupt and massive social disconnection. In addition, the otherwise insignificant relations between psychological well-being and duanzi sharing or duanzi commenting turned significantly positive when facilitated by increased bonding social capital. It is thus plausible that people who hold stronger bonding social capital were also more likely to feel psychologically better as they shared duanzi on WeChat or expressed personal opinions on duanzi with close ties outside the family household. As a result, doing so can better their psychological well-being and reinforce social connectedness with physically separated family and friends otherwise in their immediate social circles.

In sum, among the first studies to explore the social and psychological impact of the COVID-19 pandemic on individual netizens, our findings contribute to health communication and social media scholarship by: (a) identifying duanzi as a set of media-based practices that have either become a fun game among home-quarantined contacts or served socialization and resource-seeking purposes; and (b) highlighting the role of bonding and bridging social capital in a massive lockdown with unexpected public health risks. The circulation of online duanzi may convert one’s established bridging social capital into more COVID-19 knowledge or increased external social support, while a “safety net” of bonding ties mainly brings psychological comforts directly or via duanzi practices. As we focus more on the sociality mediated by the online circulation of COVID-19 related duanzi, future inquiries may take the substantive contents of these duanzi messages as well as the life stage of netizens into account, and possibly bring in a longitudinal dimension (e.g., [11,15,16]).

## Figures and Tables

**Table 1:** Categories of *duanzi*.

Category	Operational Definition	Examples
Funny anti-epidemic slogan (抗疫土味标语)	Slogans of humor and hyperbole to promote anti-epidemic campaigns.	Bring virus home to harm your parents, and you become the worst son ever. (带病回乡不孝儿郎，传染爹娘丧尽天良) Take a bite of wild animal today and meet in hell tomorrow. (今天一口野味，明天地府相会。)
Local performance	Jokes to make fun of anti-epidemic performance in local places	News: Philippine has zero cases: After its only victim passed. (菲律宾击退肺炎：确诊 1，死亡 1，治愈0) Someone arrived in Yiwu, almost believing that he is in Saudi Arabia (after seeing so many people in PE suits). (有人从义乌下车, 以为到了沙特)
Medicine jokes	Jokes to question the alleged effectiveness of anti-epidemic medicines	We buy Yan (salt) during SARS, and Shuang huang lian during COVID-19 outbreaks, for we are the offspring of Yan and Huang. (非典买盐，肺炎买双黄连，因为我们是盐黄子孙) New treatment for COVID-19: Don’t play outside household pills. (新型肺炎特效药： 不准出去丸)
Lengthy lockdown	Jokes to ridicule the length of COVID-19 lockdown	Notice: your vacation is prolonged to Feb 2; Your vacation is prolonged to Feb 10; Your vacation is prolonged to Feb 17. You need not come back to work, the company is shut off. (通知：假期延迟到 2 月 2 日，假期延迟到 2 月 10 日，假期延迟到 2 月 17 日，公司倒闭，不用回来了)。 Many restaurants and cooks are going to lose their job. People have learnt to make milk tea, steamed buns, and cakes during the lockdown. (疫情就算结束了，那些炸油条，卖凉皮，手抓饼，珍珠奶茶，做面包，蛋糕的都会失业， 因为这段时间大家都学会做饭了) Isn’t it what you always ask for? A room with good temperature, ready-made foods, WiFi access, your cell phone and nothing to do. Never thought this is coming true now. (这不是以前常说的，给你一个房间，温度合适，有食物，有手机，有网络，可以无所事事。万万没想到这有成真的一天。)

**Table 2:** Effect of duanzi practices by bonding social capital.

			Dependent Variables								
Models			Well-Being	Connected-Ness	Reduced Boredom	Well-Being	Connected-Ness	Reduced Boredom	Well-Being	Connected-Ness	Reduced Boredom
*IV’s*											
*duanzi practices*											
		Liking	−0.09	−0.17	0.29 **						
		Sharing				−0.09	0.07	0.13			
		Commenting							−0.12	−0.01	0.08
											
	Bonding social capital		0.14 *	−0.09	0.09	0.14 *	0.13	−0.03	0.12 *	0.11	−0.08
										
Interaction											
		Duanzi practices × bonding social capital	0.04	0.04	−0.07 *	0.04 *	−0.04	−0.03	0.05 *	−0.03	−0.01
										
*Control variables*											
		Age	0.00	−0.00	0.00	0.00	−0.00	0.00	0.00	−0.00	0.00
		Gender	−0.14 ***	0.03	0.02	−0.14 ***	0.03	0.02	−0.14 ***	0.02	0.02
		Education	−0.02	0.04	0.04	−0.03	0.04	0.03	−0.02 **	0.04	0.04
		Income	0.06 **	0.09 ***	−0.07 *	0.05 **	0.09 ***	−0.07 *	0.06 **	0.09 ***	−0.06 *
											
Overall model significance			*p* < 0.0001	*p* < 0.0001	*p* < 0.001	*p* < 0.0001	*p* < 0.0001	*p* < 0.05	*p* < 0.0001	*p* < 0.0001	*p* < 0.05
R^2^			0.19	0.03	0.03	0.19	0.03	0.02	0.19	0.04	0.02

* *p* ≤ 0.05; ** *p* < 0.01; *** *p* < 0.001; Moderating role of social capital.

**Table 3:** Effect of duanzi practices by bridging social capital.

Dependent Variables								
Models			External Social Support	Knowledge	External Social Support	Knowledge	External Social Support	Knowledge
*IV’s*								
	*Duanzi practices*							
		Liking	−0.04	−0.06				
		sharing			−0.28 *	−0.31 *		
		Commenting					−0.10	−0.40 **
	Bridging social capital		0.30 **	0.16	0.11	0.01	0.23 **	−0.04
*Interaction*								
		Duanzi practices × bridging SC	0.03	0.06	0.09 **	0.12 ***	0.05	0.14 ****
*Control variables*		Age	−0.00	0.00	−0.00	−0.00	−0.00	0.00
		Gender	0.02	−0.01	−0.02	−0.02	−0.01	−0.01
		Education	0.00	−0.02	−0.00	−0.03	0.01	−0.02
		Income	0.02	0.05 *	0.02	0.05 *	0.02	0.05 *
Overall model significance			*p* < 0.0001	*p* < 0.0001	*p* < 0.0001	*p* < 0.0001	*p* < 0.0001	*p* < 0.0001
R^2^			0.16	0.15	0.18	0.15	0.18	0.16

Note: Beta weights are from a final regression equation with all blocks of variables in the model. *n* = 396.* *p* <= 0.05; ** *p* < 0.01; *** *p* < 0.001; **** *p* < 0.0001 moderating role of social capital.

## Data Availability

The data presented in this study are available on request from the corresponding author.
